# *cis*-Diaqua­bis­[dimethyl (phenyl­sulfonyl­imino)­phospho­nato]cobalt(II)

**DOI:** 10.1107/S1600536811006027

**Published:** 2011-02-23

**Authors:** Elizaveta A. Trush, Victor A. Trush, Tetyana Yu. Sliva, Irina S. Konovalova, Volodymyr M. Amirkhanov

**Affiliations:** a National Taras Shevchenko University, Department of Chemistry, Volodymyrska str. 64, 01033 Kyiv, Ukraine; b STC "Institute for Syngle Crystals", 60 Lenina ave., Khar’kov 61001, Ukraine

## Abstract

In the title diaqua­cobalt complex, [Co(C_8_H_11_NO_5_PS)_2_(H_2_O)_2_], the Co^II^ atom is surrounded by six O atoms belonging to the phosphoryl and sulfonyl groups of two deprotonated chelate ligands and two additional O atoms from water mol­ecules which are in *cis* positions with respect to one another. The coordination environment of cobalt can be described as a distorted octa­hedron. O—H⋯O hydrogen bonds between the water and sulfonyl O atoms of neighboring mol­ecules form chains running parallel to [010]. Two methoxy groups attached to one phosphorus are disordered over two sets of sites in a 0.6:0.4 ratio.

## Related literature

For the coordination chemistry of β-diketone derivatives and their structural analogues, see Skopenko *et al.* (2004 ▶). For details of the pharmacological and biological properties of sulfonyl­amide derivatives, see: Kishino & Saito (1979 ▶); Xu & Angell (2000 ▶). For structural discussion, see: Cremer & Pople (1975 ▶); Zefirov *et al.* (1990 ▶). For related structures, see: Moroz *et al.* (2009 ▶); Shatrava *et al.* (2010 ▶).
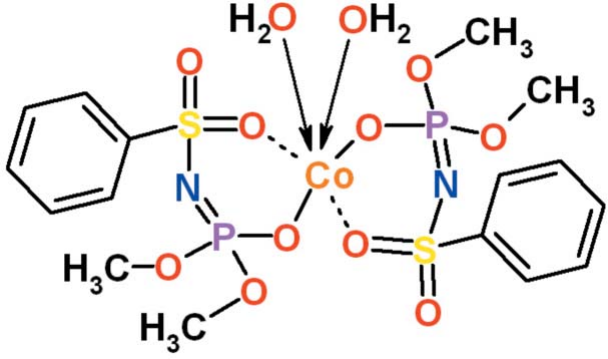


## Experimental

### 

#### Crystal data

[Co(C_8_H_11_NO_5_PS)_2_(H_2_O)_2_]
*M* *_r_* = 623.38
Triclinic, 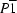

*a* = 9.875 (1) Å
*b* = 10.207 (1) Å
*c* = 13.345 (2) Å
α = 91.60 (1)°
β = 110.59 (1)°
γ = 92.84 (1)°
*V* = 1256.2 (3) Å^3^
*Z* = 2
Mo *K*α radiation
μ = 1.04 mm^−1^
*T* = 294 K
0.40 × 0.20 × 0.10 mm

#### Data collection

Oxford Diffraction Xcalibur3 diffractometer
Absorption correction: multi-scan (*CrysAlis PRO*; Oxford Diffraction, 2009 ▶) *T*_min_ = 0.681, *T*_max_ = 0.903
9231 measured reflections
5612 independent reflections
4025 reflections with *I* > 2σ(*I*)
*R*_int_ = 0.019

#### Refinement

*R*[*F* ^2^ > 2σ(*F* ^2^)] = 0.029
*wR*(*F* ^2^) = 0.066
*S* = 0.90
5612 reflections
354 parameters
4 restraints
H-atom parameters constrained
Δρ_max_ = 0.34 e Å^−3^
Δρ_min_ = −0.22 e Å^−3^

### 

Data collection: *CrysAlis CCD* (Oxford Diffraction, 2006 ▶); cell refinement: *CrysAlis RED* (Oxford Diffraction, 2006 ▶); data reduction: *CrysAlis RED*; program(s) used to solve structure: *SHELXTL* (Sheldrick, 2008 ▶); program(s) used to refine structure: *SHELXL97* (Sheldrick, 2008 ▶); molecular graphics: *ORTEPIII* (Burnett & Johnson, 1996 ▶), *ORTEP-3 for Windows* (Farrugia, 1997 ▶) and *PLATON* (Spek, 2009 ▶); software used to prepare material for publication: *SHELXL97*.

## Supplementary Material

Crystal structure: contains datablocks I, global. DOI: 10.1107/S1600536811006027/dn2630sup1.cif

Structure factors: contains datablocks I. DOI: 10.1107/S1600536811006027/dn2630Isup2.hkl

Additional supplementary materials:  crystallographic information; 3D view; checkCIF report

## Figures and Tables

**Table 1:** Hydrogen-bond geometry (Å, °)

*D*—H⋯*A*	*D*—H	H⋯*A*	*D*⋯*A*	*D*—H⋯*A*
O11—H11*A*⋯O3^i^	0.88	1.93	2.7898 (19)	167
O11—H11*B*⋯O6^i^	0.90	1.92	2.811 (2)	167
O12—H12*A*⋯O1^ii^	0.92	1.98	2.855 (2)	157
O12—H12*B*⋯O8^ii^	0.88	1.96	2.8035 (18)	163

Symmetry codes: (i) 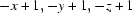; (ii) 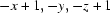.

**Fig. 1.**
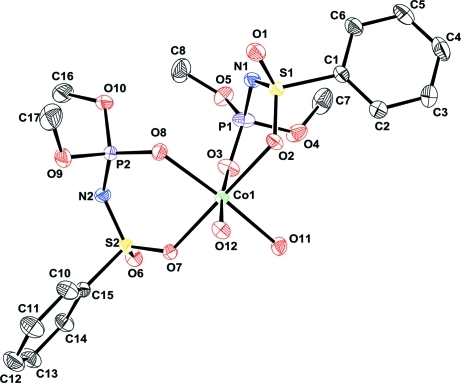
Molecular view of (I) with the atom-labelling scheme. Displacement ellipsoids are drawn at the 30% probability level. H atoms have been omitted for clarity. Only the major component of the disorder is shown in the figure.

**Fig. 2.**
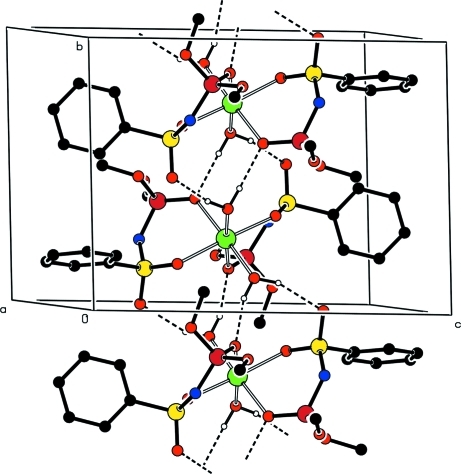
Partial packing view of compound ( I ), showing the formation of chains along [010] built from hydrogen bonds. H atoms not involved in hydrogen bonding have been omitted for clarity.

## supplementary crystallographic information

### Comment

Many efforts are devoted to the coordination chemistry of β- diketones derivatives and their structural analogues (Skopenko *et al.*, 2004). The phosphorylated sulfonylamides, RSO_2_NHP(O)(*R*')_2_ (SAPh), present such type of heterosubstituted structural analogues with different substituents at sulfur and phosphorus atoms. In the past few decades SAPh have been intensively used as bactericidal agents in medicine and toxicology (Xu & Angell, 2000). Some of them are effective pesticides (Kishino & Saito, 1979). So a variety of new s-, d-, and f- metals based coordination compounds containing this type of phosphoramides have been synthesized. Structural investigation of compounds with phosphorylated sulfonylamide ligands have already been reported (Moroz *et al.*, 2009, Shatrava *et al.*, 2010). Herein we report the structure of the title compound containing one of the simplest representative of this class of ligands: the dimethyl(phenylsulfonyl)amidophosphate.

The crystal structure of CoL_2_2H_2_O (I) is built up from non-centrosymmetric molecular species with the two water molecules in *cis-* position to each other. The CoO_6_ fragment is formed by two oxygen atoms of water molecules and four oxygen atoms of phosphoryl and sulfonyl groups from two ligands which are coordinated in bidentate chelating mode (Fig. 1). The coordination environment of cobalt can be described as a distorted octahedron.

The six-membered chelate rings have a twist–boat conformation (the puckering parameters (Cremer & Pople,1975) are θ=81.19,ψ=25.33, S=0.60 for CoO_2_SNPO_3_ fragment and θ=67.91, ψ=17.44, S=0.57 for the CoO_7_S_2_N_2_P_2_O_8_ (Zefirov *et al.*, 1990). The O4 and O5 atoms of methoxy groups are disordered over two positions due to the rotation around P1—O4 and P1—O5 bonds with populations 40:60%.

O—H···O intermolecular hydrogen bonds between the water and non-coordinated sulfonyl oxygen atoms and coordinated phosphoryl groups of neighboring SAPh molecules (Table 1) build up chains parallel to the [0 1 0] direction (Fig.2).

### Experimental

The sodium salt (NaL) was prepared by the reaction between equimolar amounts of sodium isopropylate (0,023 g, 1 mmol of Na was solved in 2-propanol) and HL (0,2652 g, 1 mmol) in an 2-propanol medium and was used for preparation of complexes without isolation from the reaction mixture.

The solution of NaL (1 mmol) was added to the solution of CoCl_2_6H_2_O (0,124 2 g, 0,5 mmol) in 2-propanol (10 ml). The resulting mixture was filtrated off and mother liquor was left on air at room temperature for several days. Precipitated from the solution purple crystals were filtered and washed with cool 2-propanol. Single crystals of [Co(*L*)_2_2H_2_O] were prepared by slow recrystallization in 2-propanol-chloroform (3:1) mixture (yield - 80–90%). This complex as prepared is less soluble in non-polar aprotic solvents and H_2_O. Analysis found: IR (KBr pellet, cm^-1^): 1220, 1060 (s, SO_2_) and 1190 (s, PO).

### Refinement

All H atoms attached to C atoms were fixed geometrically and treated as riding with C—H = 0.96 Å (methyl) or 0.93 Å (aromatic) with U_iso_(H) = 1.2U_eq_(Caromatic) or U_iso_(H) = 1.5U_eq_(Cmethyl). H atoms of water molecule were located in difference Fourier maps and included in the subsequent refinement using restraints (O-H= 0.85 (1)Å and H···H= 1.39 (2)Å) with U_iso_(H) = 1.5U_eq_(O). In the last cycles of refinement, they were treated as riding on their parent oxygen atoms.

Two methoxy groups attached to one phosphorus are disordered over two positions. Two sets of positions were then defined for the atoms of these groups and the site occupation factors of each conformation were refined while restraining their sum to unity. The site occupation factor of the major conformation refined to 0.585 (5). Then the occupancy factors were fixed to 0.6 and 0.4 respectively for the two components. The O-C distances were restrained to have chemically reasonable bond values of 1.45(0.02)Å.

### Crystal data

**Table:**

[Co(C_8_H_11_NO_5_PS)_2_(H_2_O)_2_]	*Z* = 2
*M**_r_* = 623.38	*F*(000) = 642
Triclinic, *P*1	*D*_x_ = 1.648 Mg m^−^^3^
Hall symbol: -P 1	Mo *K*α radiation, λ = 0.71073 Å
*a* = 9.875 (1) Å	Cell parameters from 4537 reflections
*b* = 10.207 (1) Å	θ = 3.0–34.8°
*c* = 13.345 (2) Å	µ = 1.04 mm^−^^1^
α = 91.60 (1)°	*T* = 294 K
β = 110.59 (1)°	Plate, purple
γ = 92.84 (1)°	0.40 × 0.20 × 0.10 mm
*V* = 1256.2 (3) Å^3^	

### Data collection

**Table:**

Oxford Diffraction Xcalibur3 diffractometer	5612 independent reflections
Radiation source: Enhance (Mo) X-ray Source	4025 reflections with *I* > 2σ(*I*)
graphite	*R*_int_ = 0.019
Detector resolution: 16.1827 pixels mm^-1^	θ_max_ = 27.5°, θ_min_ = 3.0°
ω scans	*h* = −12→12
Absorption correction: multi-scan (*CrysAlis PRO*; Oxford Diffraction, 2009)	*k* = −13→12
*T*_min_ = 0.681, *T*_max_ = 0.903	*l* = −17→17
9231 measured reflections	

### Refinement

**Table:**

Refinement on *F*^2^	Primary atom site location: structure-invariant direct methods
Least-squares matrix: full	Secondary atom site location: difference Fourier map
*R*[*F*^2^ > 2σ(*F*^2^)] = 0.029	Hydrogen site location: inferred from neighbouring sites
*wR*(*F*^2^) = 0.066	H-atom parameters constrained
*S* = 0.90	*w* = 1/[σ^2^(*F*_o_^2^) + (0.0363*P*)^2^] where *P* = (*F*_o_^2^ + 2*F*_c_^2^)/3
5612 reflections	(Δ/σ)_max_ = 0.001
354 parameters	Δρ_max_ = 0.34 e Å^−^^3^
4 restraints	Δρ_min_ = −0.22 e Å^−^^3^

### Special details

**Table:**

Experimental. Empirical absorption correction using spherical harmonics, implemented in SCALE3 ABSPACK scaling algorithm. CrysAlisPRO (Oxford Diffraction, 2009)
Geometry. All esds (except the esd in the dihedral angle between two l.s. planes) are estimated using the full covariance matrix. The cell esds are taken into account individually in the estimation of esds in distances, angles and torsion angles; correlations between esds in cell parameters are only used when they are defined by crystal symmetry. An approximate (isotropic) treatment of cell esds is used for estimating esds involving l.s. planes.
Refinement. Refinement of *F*^2^ against ALL reflections. The weighted *R*-factor *wR* and goodness of fit *S* are based on *F*^2^, conventional *R*-factors *R* are based on *F*, with *F* set to zero for negative *F*^2^. The threshold expression of *F*^2^ > σ(*F*^2^) is used only for calculating *R*-factors(gt) *etc*. and is not relevant to the choice of reflections for refinement. *R*-factors based on *F*^2^ are statistically about twice as large as those based on *F*, and *R*- factors based on ALL data will be even larger.

### Fractional atomic coordinates and isotropic or equivalent isotropic displacement parameters (Å^2^)

**Table:**

	*x*	*y*	*z*	*U*_iso_*/*U*_eq_	Occ. (<1)
Co1	0.53757 (3)	0.25048 (2)	0.49629 (2)	0.02561 (8)	
S1	0.52838 (5)	0.13796 (5)	0.26446 (4)	0.02895 (11)	
S2	0.76726 (5)	0.37652 (5)	0.72245 (4)	0.02815 (11)	
O3	0.64121 (18)	0.39270 (13)	0.43552 (12)	0.0433 (4)	
P1	0.68038 (7)	0.38043 (6)	0.33995 (5)	0.04364 (16)	
O4	0.5573 (4)	0.4744 (3)	0.2591 (3)	0.0543 (9)	0.60
C7	0.5608 (12)	0.5119 (16)	0.1582 (8)	0.076 (3)	0.60
H7A	0.6552	0.5506	0.1673	0.115*	0.60
H7B	0.4895	0.5747	0.1292	0.115*	0.60
H7C	0.5399	0.4359	0.1100	0.115*	0.60
O5	0.8157 (3)	0.4492 (2)	0.3338 (2)	0.0464 (7)	0.60
C8	0.9520 (11)	0.3888 (12)	0.3764 (11)	0.071 (3)	0.60
H8A	0.9525	0.3375	0.4358	0.106*	0.60
H8B	1.0298	0.4557	0.4001	0.106*	0.60
H8C	0.9643	0.3328	0.3216	0.106*	0.60
O4A	0.6779 (5)	0.4913 (4)	0.2748 (3)	0.0520 (11)	0.40
C7A	0.5421 (13)	0.4968 (18)	0.1864 (11)	0.074 (5)	0.40
H7A1	0.5463	0.5724	0.1462	0.112*	0.40
H7A2	0.4646	0.5029	0.2137	0.112*	0.40
H7A3	0.5253	0.4188	0.1408	0.112*	0.40
O5A	0.8642 (5)	0.3924 (4)	0.4153 (3)	0.0464 (10)	0.40
C8A	0.9676 (14)	0.3964 (12)	0.3623 (13)	0.049 (4)	0.40
H8A1	1.0639	0.4028	0.4147	0.073*	0.40
H8A2	0.9540	0.4714	0.3187	0.073*	0.40
H8A3	0.9543	0.3178	0.3178	0.073*	0.40
P2	0.86187 (6)	0.16991 (5)	0.62440 (4)	0.02887 (12)	
O1	0.57367 (17)	0.00623 (13)	0.26086 (12)	0.0414 (4)	
O2	0.45315 (15)	0.15860 (13)	0.33970 (10)	0.0321 (3)	
O6	0.79818 (17)	0.51694 (13)	0.72756 (12)	0.0384 (3)	
O7	0.61859 (15)	0.33380 (13)	0.65460 (11)	0.0341 (3)	
O8	0.71640 (15)	0.13434 (12)	0.54199 (11)	0.0334 (3)	
O9	0.91160 (17)	0.06168 (14)	0.70876 (12)	0.0436 (4)	
O10	0.97392 (16)	0.17385 (15)	0.56604 (12)	0.0413 (4)	
O11	0.36115 (15)	0.36550 (13)	0.46253 (11)	0.0370 (3)	
H11A	0.3755	0.4409	0.4997	0.055*	
H11B	0.3157	0.3944	0.3963	0.055*	
O12	0.41962 (15)	0.11126 (12)	0.54596 (11)	0.0331 (3)	
H12A	0.4369	0.0941	0.6169	0.050*	
H12B	0.3853	0.0377	0.5082	0.050*	
N1	0.65693 (18)	0.23960 (16)	0.28018 (13)	0.0342 (4)	
N2	0.88326 (18)	0.30347 (16)	0.69393 (13)	0.0335 (4)	
C1	0.3978 (2)	0.16127 (18)	0.13660 (16)	0.0307 (4)	
C2	0.2605 (3)	0.1922 (3)	0.1249 (2)	0.0519 (6)	
H2	0.2330	0.2019	0.1844	0.062*	
C3	0.1620 (3)	0.2089 (3)	0.0225 (2)	0.0660 (8)	
H3	0.0683	0.2307	0.0137	0.079*	
C4	0.2014 (3)	0.1938 (3)	−0.0644 (2)	0.0599 (7)	
H4	0.1341	0.2036	−0.1325	0.072*	
C5	0.3395 (3)	0.1643 (3)	−0.05244 (19)	0.0561 (7)	
H5	0.3667	0.1556	−0.1122	0.067*	
C6	0.4385 (3)	0.1473 (2)	0.04848 (17)	0.0458 (6)	
H6	0.5324	0.1265	0.0569	0.055*	
C10	0.7510 (3)	0.2074 (2)	0.87303 (19)	0.0443 (6)	
H10	0.7241	0.1425	0.8181	0.053*	
C11	0.7603 (3)	0.1775 (3)	0.9745 (2)	0.0555 (7)	
H11	0.7395	0.0915	0.9884	0.067*	
C12	0.7996 (3)	0.2720 (3)	1.0556 (2)	0.0576 (7)	
H12	0.8043	0.2503	1.1240	0.069*	
C13	0.8322 (3)	0.3988 (3)	1.0366 (2)	0.0566 (7)	
H13	0.8603	0.4629	1.0922	0.068*	
C14	0.8232 (3)	0.4313 (2)	0.93455 (18)	0.0430 (5)	
H14	0.8449	0.5173	0.9211	0.052*	
C15	0.7821 (2)	0.33569 (19)	0.85348 (16)	0.0306 (4)	
C16	1.1267 (3)	0.2070 (3)	0.6236 (2)	0.0603 (7)	
H16A	1.1755	0.2187	0.5734	0.090*	
H16B	1.1679	0.1373	0.6694	0.090*	
H16C	1.1380	0.2869	0.6663	0.090*	
C17	0.9315 (3)	−0.0702 (2)	0.6758 (2)	0.0691 (9)	
H17A	0.9856	−0.1161	0.7377	0.104*	
H17B	0.9836	−0.0660	0.6272	0.104*	
H17C	0.8386	−0.1159	0.6408	0.104*	

### Atomic displacement parameters (Å^2^)

**Table:**

	*U*^11^	*U*^22^	*U*^33^	*U*^12^	*U*^13^	*U*^23^
Co1	0.02756 (15)	0.02457 (13)	0.02328 (14)	0.00186 (11)	0.00724 (11)	0.00077 (10)
S1	0.0328 (3)	0.0307 (2)	0.0227 (3)	0.0018 (2)	0.0091 (2)	0.00059 (19)
S2	0.0289 (3)	0.0294 (2)	0.0243 (3)	0.0013 (2)	0.0073 (2)	−0.00163 (19)
O3	0.0625 (11)	0.0311 (7)	0.0414 (9)	−0.0096 (7)	0.0267 (8)	−0.0037 (7)
P1	0.0558 (4)	0.0341 (3)	0.0529 (4)	−0.0083 (3)	0.0358 (3)	−0.0035 (3)
O4	0.060 (2)	0.056 (2)	0.064 (2)	0.0239 (17)	0.037 (2)	0.0253 (17)
C7	0.068 (6)	0.091 (7)	0.079 (4)	0.022 (4)	0.031 (4)	0.053 (4)
O5	0.0392 (16)	0.0383 (14)	0.065 (2)	−0.0020 (12)	0.0234 (15)	0.0073 (14)
C8	0.039 (5)	0.106 (7)	0.070 (5)	0.006 (4)	0.021 (4)	0.021 (5)
O4A	0.055 (3)	0.049 (2)	0.054 (3)	−0.001 (2)	0.020 (2)	0.023 (2)
C7A	0.053 (6)	0.050 (6)	0.128 (15)	0.026 (5)	0.036 (9)	0.041 (10)
O5A	0.035 (2)	0.060 (3)	0.039 (2)	−0.011 (2)	0.010 (2)	−0.001 (2)
C8A	0.038 (6)	0.037 (5)	0.068 (8)	0.004 (4)	0.015 (5)	−0.008 (5)
P2	0.0269 (3)	0.0333 (3)	0.0255 (3)	0.0058 (2)	0.0078 (2)	0.0002 (2)
O1	0.0565 (10)	0.0318 (7)	0.0353 (9)	0.0095 (7)	0.0145 (8)	0.0011 (6)
O2	0.0348 (8)	0.0376 (7)	0.0238 (7)	−0.0052 (6)	0.0114 (6)	−0.0013 (6)
O6	0.0481 (9)	0.0289 (7)	0.0368 (8)	0.0012 (7)	0.0133 (7)	0.0019 (6)
O7	0.0268 (7)	0.0443 (8)	0.0274 (8)	0.0051 (6)	0.0053 (6)	−0.0071 (6)
O8	0.0301 (8)	0.0314 (7)	0.0341 (8)	0.0049 (6)	0.0054 (6)	−0.0053 (6)
O9	0.0526 (10)	0.0425 (8)	0.0331 (9)	0.0143 (8)	0.0102 (7)	0.0074 (7)
O10	0.0357 (9)	0.0571 (9)	0.0342 (8)	0.0069 (7)	0.0160 (7)	−0.0029 (7)
O11	0.0401 (8)	0.0308 (7)	0.0352 (8)	0.0111 (6)	0.0060 (7)	0.0028 (6)
O12	0.0417 (8)	0.0279 (7)	0.0293 (8)	−0.0029 (6)	0.0129 (7)	0.0008 (6)
N1	0.0315 (10)	0.0422 (9)	0.0309 (10)	−0.0012 (8)	0.0142 (8)	−0.0016 (8)
N2	0.0262 (9)	0.0410 (9)	0.0329 (10)	0.0008 (8)	0.0107 (8)	−0.0070 (8)
C1	0.0349 (12)	0.0289 (10)	0.0255 (10)	−0.0011 (9)	0.0077 (9)	−0.0002 (8)
C2	0.0427 (14)	0.0752 (17)	0.0370 (14)	0.0124 (13)	0.0124 (11)	−0.0053 (12)
C3	0.0429 (15)	0.093 (2)	0.0497 (16)	0.0200 (15)	0.0000 (13)	−0.0050 (15)
C4	0.070 (2)	0.0619 (16)	0.0316 (14)	0.0069 (15)	−0.0022 (13)	0.0015 (12)
C5	0.0713 (19)	0.0683 (16)	0.0274 (13)	−0.0002 (15)	0.0165 (13)	0.0006 (12)
C6	0.0444 (14)	0.0629 (14)	0.0303 (12)	0.0001 (12)	0.0140 (11)	−0.0003 (11)
C10	0.0556 (15)	0.0395 (12)	0.0403 (14)	−0.0070 (11)	0.0218 (12)	−0.0029 (10)
C11	0.0700 (18)	0.0553 (15)	0.0483 (16)	−0.0082 (14)	0.0309 (14)	0.0101 (13)
C12	0.0610 (18)	0.0813 (19)	0.0353 (14)	−0.0014 (15)	0.0235 (13)	0.0094 (14)
C13	0.0611 (17)	0.0721 (17)	0.0324 (13)	−0.0076 (14)	0.0142 (12)	−0.0139 (12)
C14	0.0498 (14)	0.0428 (12)	0.0333 (12)	−0.0065 (11)	0.0126 (11)	−0.0065 (10)
C15	0.0260 (10)	0.0380 (11)	0.0267 (11)	0.0014 (9)	0.0081 (9)	0.0000 (9)
C16	0.0355 (14)	0.087 (2)	0.0619 (18)	0.0025 (14)	0.0220 (13)	−0.0037 (15)
C17	0.085 (2)	0.0417 (14)	0.075 (2)	0.0230 (14)	0.0182 (17)	0.0126 (14)

### Geometric parameters (Å, °)

**Table:**

Co1—O12	2.0608 (13)	P2—O10	1.5607 (16)
Co1—O11	2.0729 (13)	P2—O9	1.5695 (15)
Co1—O3	2.0771 (14)	P2—N2	1.5897 (17)
Co1—O8	2.0942 (13)	O9—C17	1.448 (3)
Co1—O7	2.1143 (14)	O10—C16	1.450 (3)
Co1—O2	2.1282 (14)	O11—H11A	0.8808
S1—O1	1.4427 (14)	O11—H11B	0.9049
S1—O2	1.4604 (14)	O12—H12A	0.9239
S1—N1	1.5497 (18)	O12—H12B	0.8753
S1—C1	1.771 (2)	C1—C2	1.363 (3)
S2—O6	1.4452 (14)	C1—C6	1.376 (3)
S2—O7	1.4646 (14)	C2—C3	1.392 (3)
S2—N2	1.5454 (17)	C2—H2	0.9300
S2—C15	1.766 (2)	C3—C4	1.353 (4)
O3—P1	1.4610 (16)	C3—H3	0.9300
P1—O4A	1.442 (4)	C4—C5	1.367 (4)
P1—O5	1.508 (3)	C4—H4	0.9300
P1—N1	1.5903 (18)	C5—C6	1.380 (3)
P1—O4	1.677 (4)	C5—H5	0.9300
P1—O5A	1.735 (4)	C6—H6	0.9300
O4—C7	1.422 (7)	C10—C11	1.369 (3)
C7—H7A	0.9600	C10—C15	1.383 (3)
C7—H7B	0.9600	C10—H10	0.9300
C7—H7C	0.9600	C11—C12	1.365 (4)
O5—C8	1.442 (8)	C11—H11	0.9300
C8—H8A	0.9600	C12—C13	1.370 (4)
C8—H8B	0.9600	C12—H12	0.9300
C8—H8C	0.9600	C13—C14	1.384 (3)
O4A—C7A	1.446 (9)	C13—H13	0.9300
C7A—H7A1	0.9600	C14—C15	1.371 (3)
C7A—H7A2	0.9600	C14—H14	0.9300
C7A—H7A3	0.9600	C16—H16A	0.9600
O5A—C8A	1.432 (10)	C16—H16B	0.9600
C8A—H8A1	0.9600	C16—H16C	0.9600
C8A—H8A2	0.9600	C17—H17A	0.9600
C8A—H8A3	0.9600	C17—H17B	0.9600
P2—O8	1.4899 (14)	C17—H17C	0.9600
			
O12—Co1—O11	87.46 (6)	H8A1—C8A—H8A3	109.5
O12—Co1—O3	175.32 (6)	H8A2—C8A—H8A3	109.5
O11—Co1—O3	89.17 (6)	O8—P2—O10	107.45 (8)
O12—Co1—O8	90.37 (5)	O8—P2—O9	112.00 (9)
O11—Co1—O8	175.81 (6)	O10—P2—O9	105.32 (8)
O3—Co1—O8	93.20 (6)	O8—P2—N2	118.12 (8)
O12—Co1—O7	88.52 (6)	O10—P2—N2	108.42 (9)
O11—Co1—O7	89.44 (5)	O9—P2—N2	104.80 (9)
O3—Co1—O7	94.69 (6)	S1—O2—Co1	128.04 (8)
O8—Co1—O7	86.92 (5)	S2—O7—Co1	129.91 (9)
O12—Co1—O2	88.98 (5)	P2—O8—Co1	126.80 (8)
O11—Co1—O2	91.24 (6)	C17—O9—P2	120.67 (16)
O3—Co1—O2	87.86 (6)	C16—O10—P2	121.54 (14)
O8—Co1—O2	92.30 (5)	Co1—O11—H11A	116.3
O7—Co1—O2	177.37 (5)	Co1—O11—H11B	122.1
O1—S1—O2	113.80 (9)	H11A—O11—H11B	98.9
O1—S1—N1	110.36 (10)	Co1—O12—H12A	124.1
O2—S1—N1	113.78 (9)	Co1—O12—H12B	121.0
O1—S1—C1	106.44 (9)	H12A—O12—H12B	107.1
O2—S1—C1	105.01 (9)	S1—N1—P1	125.91 (11)
N1—S1—C1	106.78 (9)	S2—N2—P2	127.90 (11)
O6—S2—O7	113.82 (9)	C2—C1—C6	120.5 (2)
O6—S2—N2	110.57 (9)	C2—C1—S1	121.42 (17)
O7—S2—N2	113.34 (9)	C6—C1—S1	118.12 (17)
O6—S2—C15	105.87 (9)	C1—C2—C3	119.0 (2)
O7—S2—C15	105.32 (9)	C1—C2—H2	120.5
N2—S2—C15	107.28 (9)	C3—C2—H2	120.5
P1—O3—Co1	127.44 (9)	C4—C3—C2	120.6 (3)
O4A—P1—O3	120.9 (2)	C4—C3—H3	119.7
O4A—P1—O5	56.9 (2)	C2—C3—H3	119.7
O3—P1—O5	121.88 (14)	C3—C4—C5	120.3 (2)
O4A—P1—N1	115.9 (2)	C3—C4—H4	119.8
O3—P1—N1	117.82 (9)	C5—C4—H4	119.8
O5—P1—N1	108.73 (12)	C4—C5—C6	119.8 (2)
O4A—P1—O4	41.9 (2)	C4—C5—H5	120.1
O3—P1—O4	99.20 (13)	C6—C5—H5	120.1
O5—P1—O4	98.84 (17)	C1—C6—C5	119.7 (2)
N1—P1—O4	106.62 (15)	C1—C6—H6	120.1
O4A—P1—O5A	98.0 (2)	C5—C6—H6	120.1
O3—P1—O5A	92.25 (16)	C11—C10—C15	118.9 (2)
O5—P1—O5A	42.78 (16)	C11—C10—H10	120.5
N1—P1—O5A	103.27 (16)	C15—C10—H10	120.5
O4—P1—O5A	137.9 (2)	C12—C11—C10	121.0 (2)
C7—O4—P1	122.6 (6)	C12—C11—H11	119.5
O4—C7—H7A	109.5	C10—C11—H11	119.5
O4—C7—H7B	109.5	C11—C12—C13	120.1 (2)
H7A—C7—H7B	109.5	C11—C12—H12	119.9
O4—C7—H7C	109.5	C13—C12—H12	119.9
H7A—C7—H7C	109.5	C12—C13—C14	119.9 (2)
H7B—C7—H7C	109.5	C12—C13—H13	120.1
C8—O5—P1	119.5 (5)	C14—C13—H13	120.1
O5—C8—H8A	109.5	C15—C14—C13	119.4 (2)
O5—C8—H8B	109.5	C15—C14—H14	120.3
H8A—C8—H8B	109.5	C13—C14—H14	120.3
O5—C8—H8C	109.5	C14—C15—C10	120.66 (19)
H8A—C8—H8C	109.5	C14—C15—S2	119.84 (16)
H8B—C8—H8C	109.5	C10—C15—S2	119.49 (16)
P1—O4A—C7A	113.0 (7)	O10—C16—H16A	109.5
O4A—C7A—H7A1	109.5	O10—C16—H16B	109.5
O4A—C7A—H7A2	109.5	H16A—C16—H16B	109.5
H7A1—C7A—H7A2	109.5	O10—C16—H16C	109.5
O4A—C7A—H7A3	109.5	H16A—C16—H16C	109.5
H7A1—C7A—H7A3	109.5	H16B—C16—H16C	109.5
H7A2—C7A—H7A3	109.5	O9—C17—H17A	109.5
C8A—O5A—P1	119.7 (7)	O9—C17—H17B	109.5
O5A—C8A—H8A1	109.5	H17A—C17—H17B	109.5
O5A—C8A—H8A2	109.5	O9—C17—H17C	109.5
H8A1—C8A—H8A2	109.5	H17A—C17—H17C	109.5
O5A—C8A—H8A3	109.5	H17B—C17—H17C	109.5

### Hydrogen-bond geometry (Å, °)

**Table:**

*D*—H···*A*	*D*—H	H···*A*	*D*···*A*	*D*—H···*A*
O11—H11A···O3^i^	0.88	1.93	2.7898 (19)	167
O11—H11B···O6^i^	0.90	1.92	2.811 (2)	167
O12—H12A···O1^ii^	0.92	1.98	2.855 (2)	157
O12—H12B···O8^ii^	0.88	1.96	2.8035 (18)	163

Symmetry codes: (i) −*x*+1, −*y*+1, −*z*+1; (ii) −*x*+1, −*y*, −*z*+1.
